# Inhibition of Catalase by Tea Catechins in Free and Cellular State: A Biophysical Approach

**DOI:** 10.1371/journal.pone.0102460

**Published:** 2014-07-15

**Authors:** Sandip Pal, Subrata Kumar Dey, Chabita Saha

**Affiliations:** Department of Biotechnology, West Bengal University of Technology, Kolkata, West Bengal, India; University of Quebect at Trois-Rivieres, Canada

## Abstract

Tea flavonoids bind to variety of enzymes and inhibit their activities. In the present study, binding and inhibition of catalase activity by catechins with respect to their structure-affinity relationship has been elucidated. Fluorimetrically determined binding constants for (−)-epigallocatechin gallate (EGCG) and (−)-epicatechin gallate (ECG) with catalase were observed to be 2.27×10^6^ M^−1^ and 1.66×10^6^ M^−1^, respectively. Thermodynamic parameters evidence exothermic and spontaneous interaction between catechins and catalase. Major forces of interaction are suggested to be through hydrogen bonding along with electrostatic contributions and conformational changes. Distinct loss of α-helical structure of catalase by interaction with EGCG was captured in circular dichroism (CD) spectra. Gallated catechins demonstrated higher binding constants and inhibition efficacy than non-gallated catechins. EGCG exhibited maximum inhibition of pure catalase. It also inhibited cellular catalase in K562 cancer cells with significant increase in cellular ROS and suppression of cell viability (IC_50_ 54.5 µM). These results decipher the molecular mechanism by which tea catechins interact with catalase and highlight the potential of gallated catechin like EGCG as an anticancer drug. EGCG may have other non-specific targets in the cell, but its anticancer property is mainly defined by ROS accumulation due to catalase inhibition.

## Introduction

**Figure 1:**
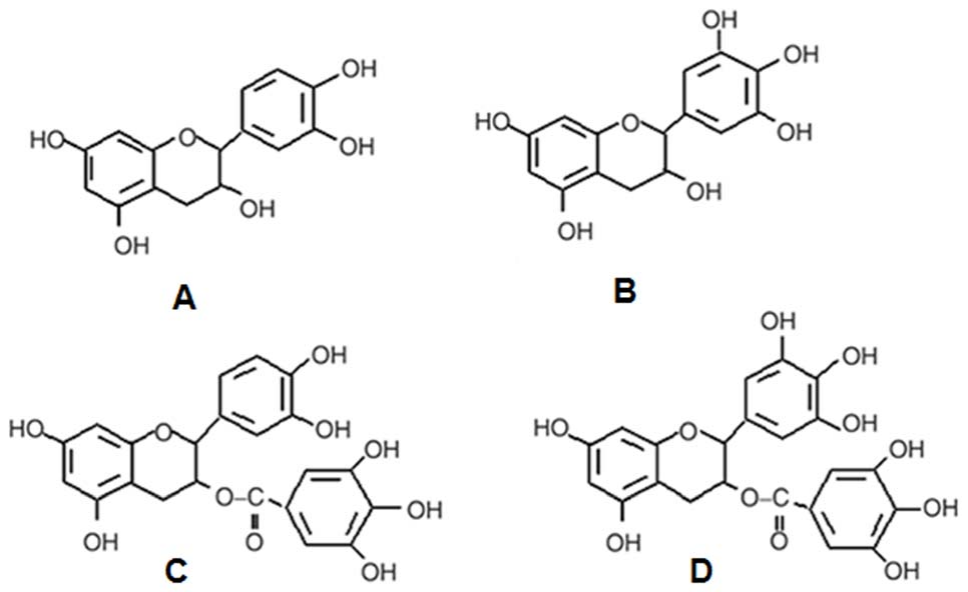
Chemical structures of four catechins: (A) EC, (B) EGC, (C) ECG, and (D) EGCG.

Green tea polyphenols have received wide attention for their beneficial health effects. Catechins have been effective for cancer prevention studies [1]–[3]. The major catechins copiously present in tea extract, especially in green tea, are (−)-epicatechin (EC), (−)-epigallocatechin (EGC), (−)-epicatechin gallate (ECG), and (−)-epigallocatechin gallate (EGCG) as illustrated in Figure 1. The major anticancer activities of tea catechins are as antioxidants, pro-oxidants and enzyme inhibitors [4]–[8]. Antioxidant activity of tea polyphenols has found wide application in radioprotection and chemoprevention by scavenging reactive oxygen species (ROS) [9]–[13]. Galloylated catechins, especially EGCG, are known to inhibit growth of cancer cells and induce apoptosis in various types of tumor cells due to their pro-oxidant activity [14]–[17]. Various mechanisms may be associated with the pro-oxidant behavior of flavonoids in cancer cells, of which enzyme inhibition is a major process.

The ability of flavonoids to bind and inhibit some vital cellular enzymes leading to suppression of cell proliferation is being investigated widely [18]–[22]. EGCG is known to bind to proteins like salivary proline-rich proteins, fibronectin, fibrinogen and histidine rich glycoproteins [23]. Caseins and lactoglobulins are milk proteins which rendered the antioxidant activity of tea polyphenols upon binding with catechins [24]–[27]. EGCG binds to Bcl-2 proteins with inhibition constant (*K*_i_) 0.33–0.49 µM and to vimentin and G3BP1 with dissociation constant (*K*_d_) 3.3 nM and 0.4 µM, respectively [28]–[30]. In the present study, binding of catechins to catalase – an enzyme which maintains the cellular ROS levels is followed fluorimetrically and thermodynamically characterized.

Catalase is an antioxidant oligomeric enzyme (MW 2,40,000) with four identical subunits arranged tetrahedrally [31]–[33]. Each subunit consists of a single polypeptide chain that associates with a prosthetic group, ferric protoporphyrin IX [34]. It is a very important enzyme in protecting cells from oxidative damage by ROS. Drugs bind to enzymes and elicit enzyme inhibition; one such example is catalase inhibition by wogonin led to H_2_O_2_ accumulation and cytotoxicity in cancer cells through H_2_O_2_-mediated NF-κB suppression and apoptosis activation [35]. Similarly, EGCG at concentrations between 5–20 µM also inhibited phosphorylation of JNK, JUN, MEK1, MEK2 in JB6 epidermal cell lines [36]. In MCF7 breast cancer cell lines, cyclin dependent kinase 2 (CDK2) and CDK4 were reported to be inhibited by 30 µM EGCG [37]. In KYSE 510 human esophageal cancer cells, EGCG inhibited DNA methyltransferase with *K*_i_ 7 µM [38]. All these enzymes have relevance in cancer prevention. Inhibition of human cancer cell growth by tea polyphenols has been observed in H1299, H661, HT-29, H441 and breast cancer cell lines at IC_50_ values between (20–75 µg/ml) [39]. Catalase is another cellular enzyme whose interaction with microsystin, a cyanotoxin drug, decreases its enzymatic action [40]. Here its inhibition in K562 cancer cells is reported and its relevance in cancer is demonstrated by the suppression of cell viability. The study also highlights the influence of structure of the catechins on the inhibition of catalase activity by catechins.

As endogenously formed ROS are important in promoting carcinogenesis, tea polyphenols may have important role in quenching them and also tea polyphenols being redox active can under go auto-oxidation and produce ROS in media and in mitochondria [41], [42]. This makes activity of tea polyphenols in cancer prevention very complex which needs active investigation. Changes in ROS population are expected manifestation of catalase inhibition and have been followed to monitor the enzyme activity on EGCG treatment.

## Materials and Methods

### Chemicals

Catalase (from bovine liver), EC, ECG, EGC, EGCG, dimethyl sulfoxide (DMSO), 2′,7′-dichloroflurescein diacetate (DCFDA) were obtained from Sigma-Aldrich, St. Louis, MO, USA. 3-amino-1,2,4-triazole (3-AT), 3-(4,5-dimethylthiazol-2-yl)-2, 5-diphenyltetrazolium bromide (MTT) were procured from Himedia, Mumbai, India. RPMI 1640 media, fetal bovine serum (FBS), penicillin-streptomycin were obtained from Gibco, Grand Island, NY. All the experiments were carried out in 50 mM phosphate buffer of pH 7.4.

### Cell culture

K562 (human chronic myeloid leukemia cell line) was procured from NCCS, Pune and grown in RPMI 1640 supplemented with 10% FBS, 100 U/ml of penicillin and 100 µg/ml of streptomycin in 25 cm^2^ T-flasks (Nunc, Roskilde, Denmark) with vented caps and incubated at 37°C in a humidified atmosphere of 5% CO_2_ in air.

### Fluorescence spectroscopy

Fluorescence spectra were recorded using a Perkin Elmer fluorescence spectrophotometer (LS-55) equipped with 150 W Xenon flash lamp and using fluorescence-free quartz cell of 1 cm path length. The widths of excitation and emission slits were set to 5 nm, and scan speed (100 nm/min), excitation voltage were kept constant for each data set. Quantitative analysis of the potential interaction between catechins and catalase was performed by the fluorimetric titration technique. Briefly, solution of catalase (5×10^−7^ M, as calculated using molar extinction coefficient of 3.24×10^5^ M^−1^ s^−1^ at 405 nm for catalase) in 50 mM phosphate buffer of pH 7.4 was titrated in cuvette by successive additions of individual catechin solution aliquots from a stock of 5×10^−7 ^M. Fluorescence emission spectra were recorded in the wavelength range of 290–450 nm upon excitation at 280 nm when catalase samples were titrated with catechins. All experiments were carried out at room temperature. Fluorescence intensity was measured at 340 nm of protein emission spectra. Fluorescence spectra of individual catechins in buffer were recorded as blanks under the same experimental conditions and subtracted from the corresponding sample to correct the fluorescence background.

### Isothermal titration calorimetry (ITC)

The energetics of the binding of catechins to catalase was studied by ITC using a MicroCal ITC_200_, (Northampton, MA, USA). All solutions were degassed under vacuum (140 mbar, 10 min) on the MicroCal’s Thermovac unit to eliminate air bubble formation inside the calorimeter cell. Briefly, the calorimeter syringe was filled with a concentrated solution of catechins (10 µM each). Successive injections of this solution into 1 µM solution of catalase in the calorimeter cell were effected from the rotating syringe with constant stirring of the solution. The titration and analysis were performed through Origin 7 software provided with the unit.

### Circular dichroism (CD) spectroscopy

All the CD experiments were carried out on a Jasco-815 automatic spectropolarimeter (Jasco International Co., Ltd. Hachioji, Japan) equipped with a peltier cuvette holder and temperature controller PFD425 L/15. The catalase concentration and path length of the cuvette used were 1 µM and 0.1 cm, respectively. The instrument parameters were set at scanning speed of 50 nm/min, bandwidth of 1.0 nm and sensitivity of 100 milli degree. The molar ellipticity values are expressed in terms of mean residue molar ellipticity, in units of deg. cm^2^ dmol^−1^.

### Inhibition of catalase activity

Absorption spectrophotometer (Varian, CARY 100 Bio, USA) was used to determine pure catalase activity (cell free). Briefly, 900 µl of H_2_O_2_ (0.036%) was taken in quartz cell of 1 cm path length and its absorbance was recorded at 240 nm. To the solution, 100 µl of catalase (50 U/ml) was added and the decrease in absorbance was recorded at 10 s interval. This experiment was performed in presence 50 µM each of four catechins and 3-AT separately to evaluate the inhibitory effect of catechins on catalase activity, after 1 h incubation.

Estimation of cellular catalase activity was carried out by suspending K562 cells (5×10^4^ for each sample) in 1 ml of PBS along with four catechins and 3-AT (50 µM each) separately and incubated at 37°C for 2 hours. Later, each sample was centrifuged and washed twice with PBS. The final pellet was suspended in 200 µl lysis buffer and kept on ice for 30 min. The lysate was then used in the same protocol used for evaluation of pure catalase activity.

### Analysis of cell viability

Cell viability of K562 cells was quantified by MTT based colorimetric assay as described elsewhere [43]. Briefly, cells were rinsed with PBS and 0.5 mg/ml MTT was added into each sample. The mixture was incubated at 37°C for an additional 3 h. At the end of incubation period, the medium containing MTT was removed and the pellet was dissolved in 200 µl DMSO. Absorbance was measured at a wavelength of 570 nm. Cell viability was expressed as a percentage of the control culture.

### Measurement of intracellular ROS

Intracellular ROS were estimated by using the DCFDA fluorescent probe [9]. Intracellular H_2_O_2_ and other peroxides can oxidize DCFDA to highly fluorescent compound dichlorofluorescein (DCF). K562 cells were incubated at 37°C in absence and presence of EGCG (12.5 – 100 µM) for 24 h. Cells were again incubated with 10 µM DCFDA at 37°C for an additional 30 min, and then washed twice with PBS. Finally the fluorescence intensity of DCF was measured with an excitation wavelength of 485 nm and emission wavelength of 530 nm.

### Statistical analysis

Data were analyzed by Origin Software (Version 8) for Windows. The statistical analysis of the samples was undertaken using Student’s *t*-test. All data reported are means ± standard deviations for three independent experiments, unless otherwise noted.

## Results

### Quenching of catalase fluorescence by catechins

**Figure 2:**
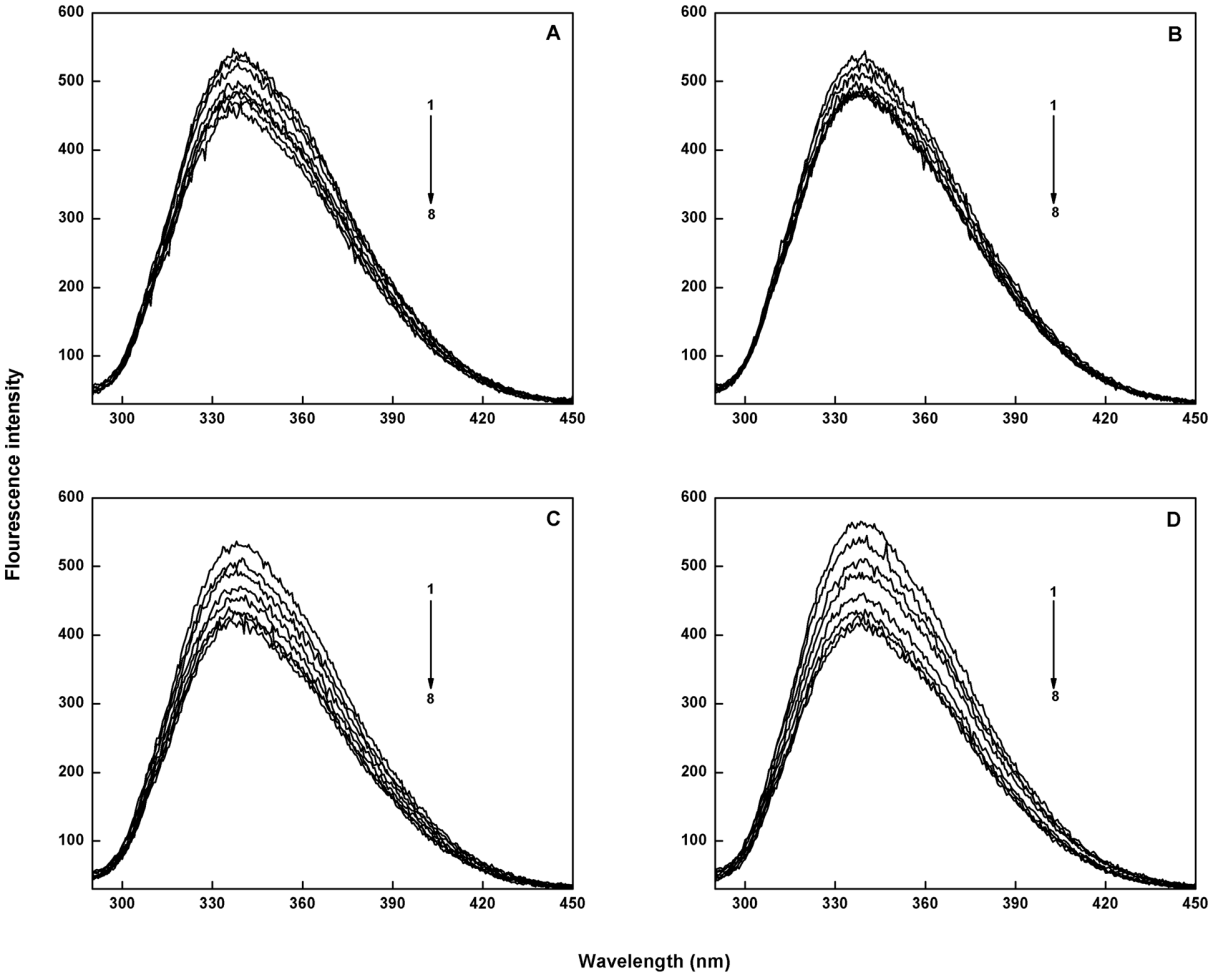
The fluorescence emission spectra of native catalase (5×10^−7^ M) and the quenching effect of catechins on its fluorescence intensity at 25°C (λ_ex_ = 280 nm): (A) EC-catalase, (B) EGC-catalase, (C) ECG-catalase, and (D) EGCG-catalase. Curves (1 to 8) denote 2.5×10^−9^ M to 1.7×10^−8^ M of catechins.

**Figure 3:**
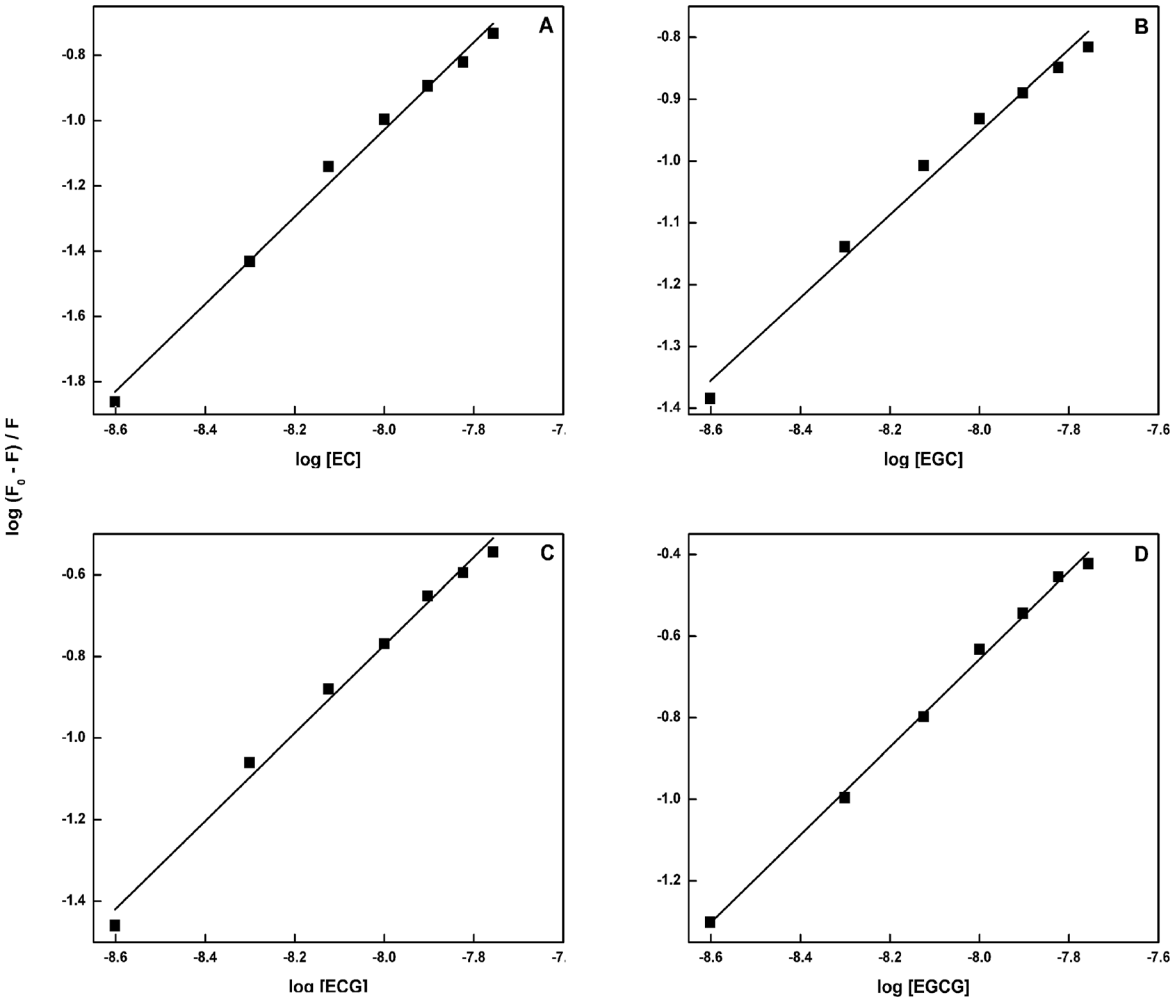
Double logarithmic plot of catalase-catechins system at 25°C (λ_ex_ = 280 nm). (A) EC-catalase, (B) EGC-catalase, (C) ECG-catalase, and (D) EGCG-catalase.

Catalase shows a fluorescence emission peak at 340 nm with the excitation wavelength at 280 nm, mainly due to the presence of tryptophan and tyrosine residues [44]. This emission peak exhibited progressive decrease in intensity on addition of catechins suggesting interaction between catalase and catechins (Figure 2 A–D). The binding constants and the number of binding sites involved were calculated according to the relation: log (F_0_–F)/F = log K+n log [Q], where K is the binding constant and n is the number of binding sites, F_0_ is the fluorescence intensity of free catalase, F is the consecutive fluorescence on addition of catechins, and [Q] is the concentration of the quencher (here catechins). A plot of log (F_0_– F)/F and log [Q] was used to determine the values of K and n from the intercept and the slope respectively (Figure 3 A–D). The binding constants of EC, EGC, ECG and EGCG with catalase are calculated to be 1.08×10^5^, 7.9×10^4^, 1.66×10^6^, and 2.27×10^6^ M^−1^, respectively with single binding site.

### Calorimetric characterization of catalase-catechins interaction

**Figure 4:**
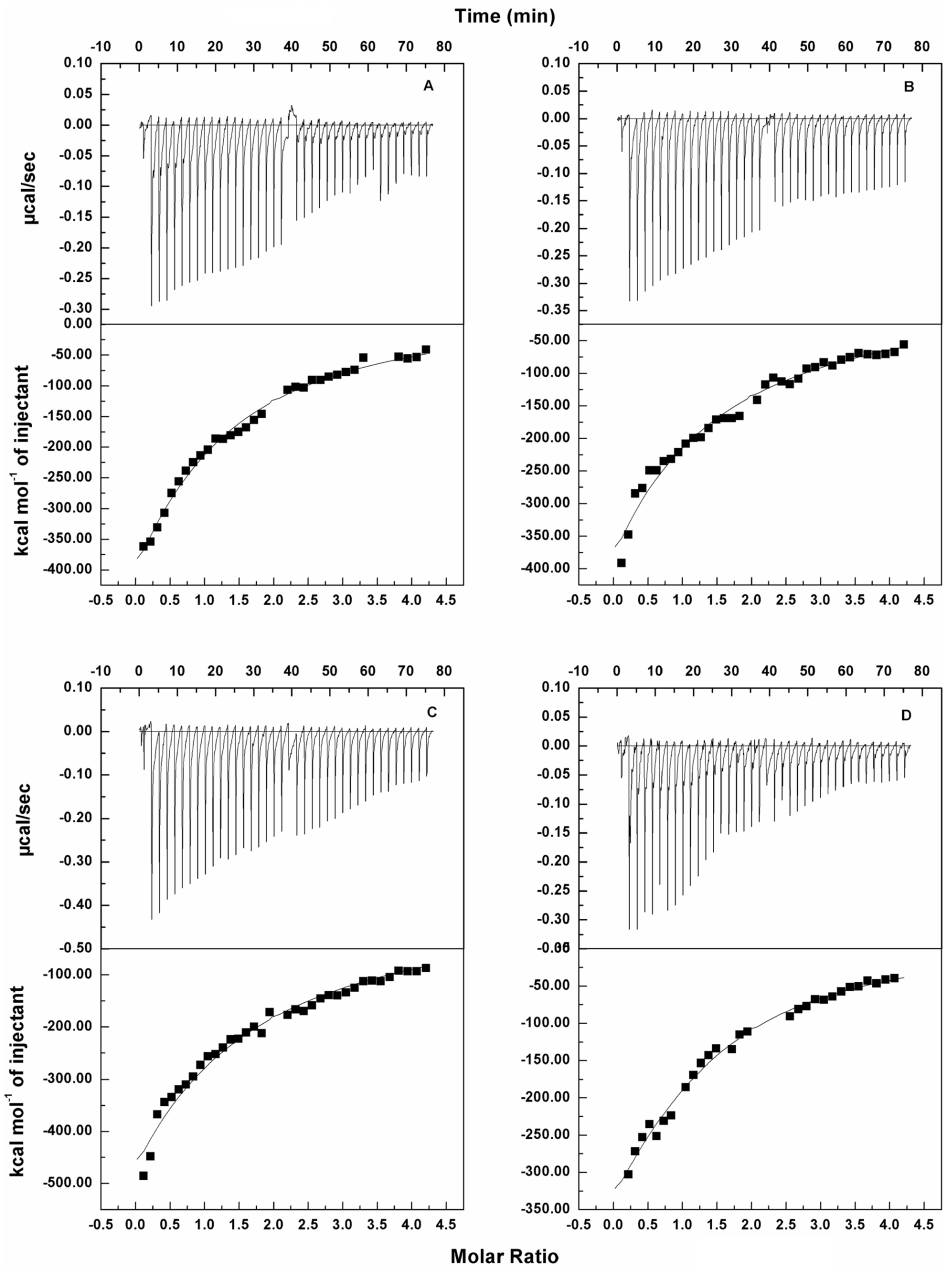
ITC profiles for catalase (1 µM) when titrated with catechins (10 µM each) at 25°C: (A) EC-catalase, (B) EGC-catalase, (C) ECG-catalase, and (D) EGCG-catalase systems.

ITC directly measures the heat released during a chemical reaction. ITC can reveal the stoichiometry, enthalpy free energy, and entropy changes that occur over the course of a reaction [45], [46]. Data obtained from the titration are presented by the series of peaks corresponding to each injection. Usually, this representation is transformed into the apparent heat change between two injections as a function of the titrant ratio by means of integration of power differential with regard to time. ITC has been used mainly to quantify interactions in various biochemical systems [47]–[49]. ITC has been used in the present study to determine binding affinity and other thermodynamic parameters of catechins-catalase interaction. Representative isothermal calorimetric heat profiles for the binding of catechins-catalase interaction, at physiological pH and 25°C are shown in Figure 4 (A–D). The data were corrected for the heat of dilution of catechins, which was determined in a separate set of experiments under identical conditions. The titration and analysis were performed using one-site binding model through Origin 7 software provided with the unit. The thermodynamic parameters recorded for the binding of catechins (10 µM each) with catalase (1 µM) are summarized in Table 1. From Figure 4 it is observed that the titration of catalase with all the four catechins yield negative heat deflections. EC, EGC, ECG, and EGCG bind to catalase with binding constants 2.13×10^5^, 1.92×10^5^, 6.36×10^5^, and 8.19×10^5^ M^−1^, respectively, as determined by the following equation: Δ*G* = −*RT ln K*, where Δ*G* is the free energy, *R* and *T* are the gas constant and temperature, respectively, *K* is the binding constant.

**Table 1:** Thermodynamic parameters from ITC experiments for catalase-catechins system at 25°C.

System	Δ*G* (cal/mol)^a^	Δ*H* (cal/mol)^b^	Δ*S* (cal/mol/K)^b^
**EGCG – Catalase**	−7.95×10^3^	−4.50×10^5^±2.25×10^4^	−1.48×10^3^
**ECG – Catalase**	−7.80×10^3^	−7.56×10^5^±3.02×10^4^	−2.60×10^3^
**EC – Catalase**	−7.17×10^3^	−7.31×10^9^±2.15×10^8^	−2.40×10^7^
**EGC – Catalase**	−7.10×10^3^	−3.20×10^9^±1.06×10^8^	−1.07×10^7^

a Calculated from the average values of Δ*H*.

b Directly extracted from the experiment.

### Catechin induced conformational changes in catalase

**Figure 5:**
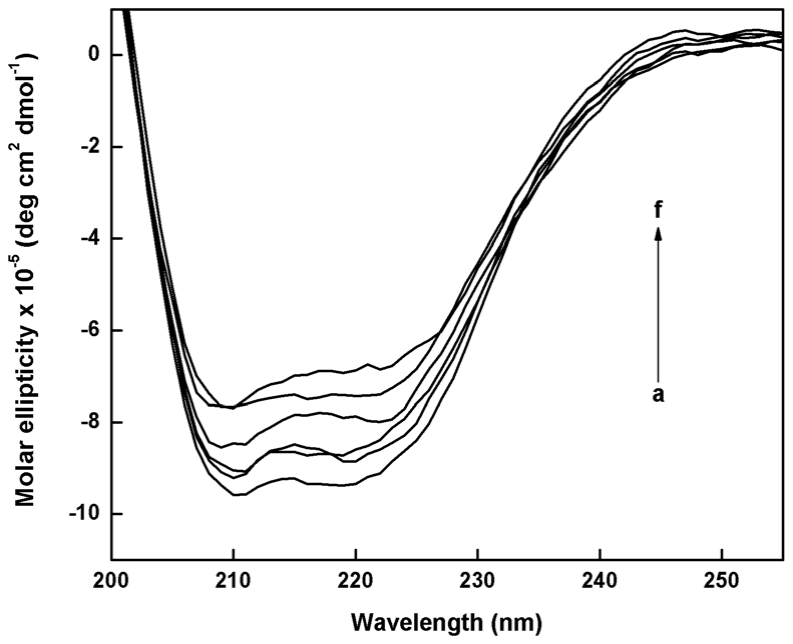
The CD spectra of catalase (1 µM) and its complexes with catechins and 3-AT (3 µM each) at 25°C. The curves (a to f) represent pure catalase, EGC-catalase, EC-catalase, ECG-catalase, EGCG-catalase, and 3-AT-catalase complexes, respectively.

CD allows investigation of the conformational changes that occur in a protein upon ligand binding [50]. The CD spectra of catalase at pH 7.4 in the absence and in the presence of EGC, EC, ECG, EGCG, and 3-AT at 25°C are represented in Figure 5 (a – f). The CD spectra of catalase exhibited two negative bands in the far-UV region at 208 and 220 nm, characteristic of an α-helical structure of protein [51]. The negative band at 208 and 220 nm decreased in intensity with the addition of various catechins, which is indicative of the loss of α-helicity upon interaction. The CD spectra of catalase in presence and absence of catechins were observed to be similar in shape, indicating that the structure of catalase is predominantly α-helical [52]. From the CD spectra, the effect of catechins on the secondary structure of catalase is in the following order: 3-AT>EGCG>ECG>EC>EGC.

### Inhibition of catalase activity

**Figure 6:**
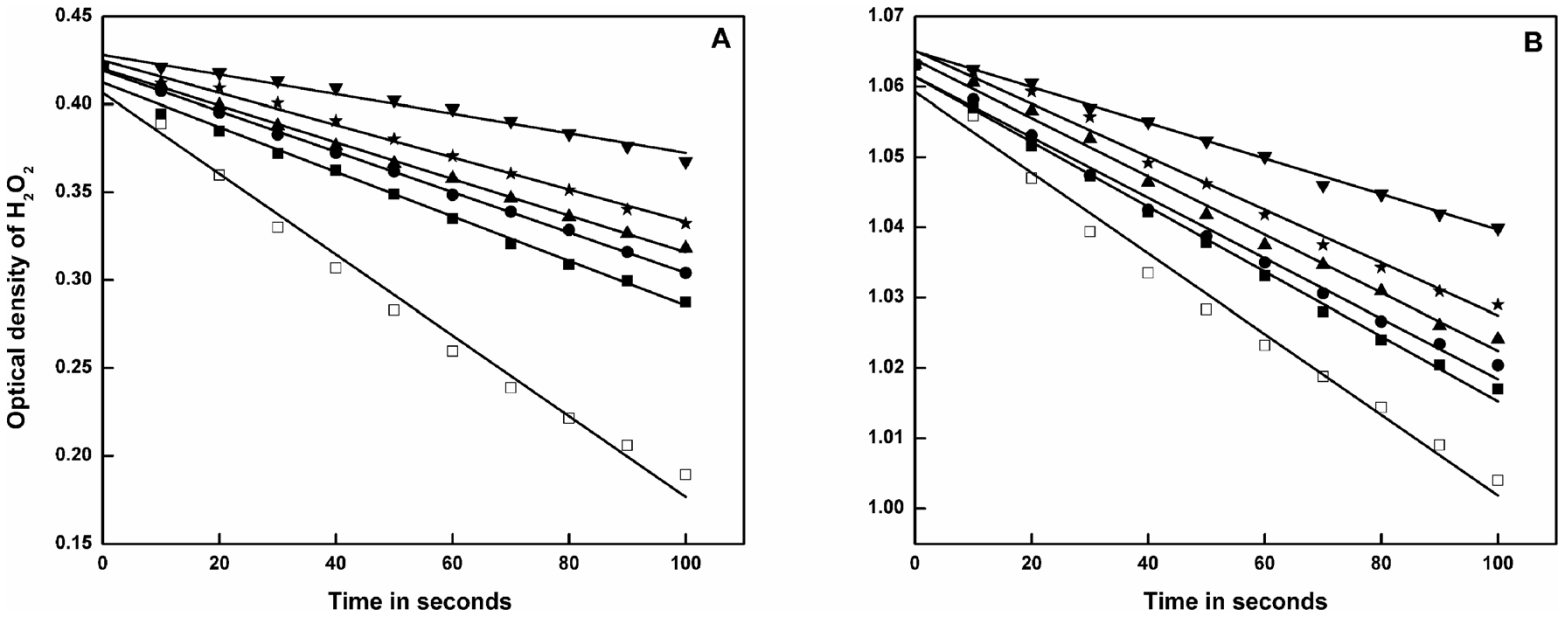
Decay curves of H_2_O_2_ by pure catalase (A) and by cellular catalase (B) in absence (□) and presence of EGC (▪), EC (•), ECG (▴), EGCG (*), 3-AT (▾) as recorded by spectrophotometer at 240 nm. Initial H_2_O_2_ concentration was approximately 0.01 mM.

**Figure 7:**
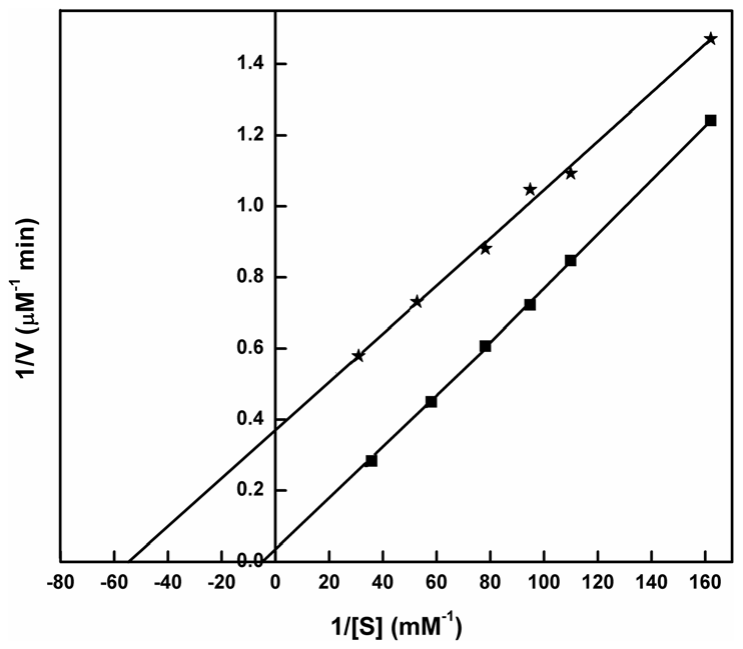
Double reciprocal plot or Lineweaver-Burk plot of catalase – H_2_O_2_ (▪) and catalase+EGCG – H_2_O_2_ (*) reactions.

The spectroscopic approach for determination of catalase activity on H_2_O_2_ degradation showed continuous breakdown of its substrate, H_2_O_2_ with time (upto 100 s) at 240 nm. On addition of catechins, a decrease in rate of H_2_O_2_ degradation i.e. a decrease in pure catalase activity was observed which is represented in Figure 6A. Catechins with galloyl moiety (ECG and EGCG) reduced catalase activity more than its non-gallated counterparts i.e. EGC and EC. However, Inhibition by EGCG is the closest to inhibition by known catalase inhibitor 3-AT. The order of inhibition of catalase activity was found to be 3-AT>EGCG>ECG>EC>EGC. The same experiment with cellular catalase in K562 cells revealed inhibition of cellular catalase activity in the presence of catechins (Figure 6B). The trend of inhibition in this condition was observed to be similar to that of pure catalase. The double reciprocal plot or Lineweaver-Burk plot shows that the inhibition of catalase by EGCG is of uncompetitive type (Figure 7). The y-intercept i.e. 1/V_0_ value for uninhibited and inhibited catalase were found to be 0.0081 and 0.3635 µM^−1^ min, respectively.

### MTT assay for cell viability

The concentration (IC_50_) at which the K562 cell survivability was reduced to 50% was calculated for EGCG and 3-AT by MTT based cell viability assay. The IC_50_ value obtained for EGCG and 3-AT was 54.5 µM and 10.5 µM, respectively.

### Measurement of intracellular ROS

**Figure 8:**
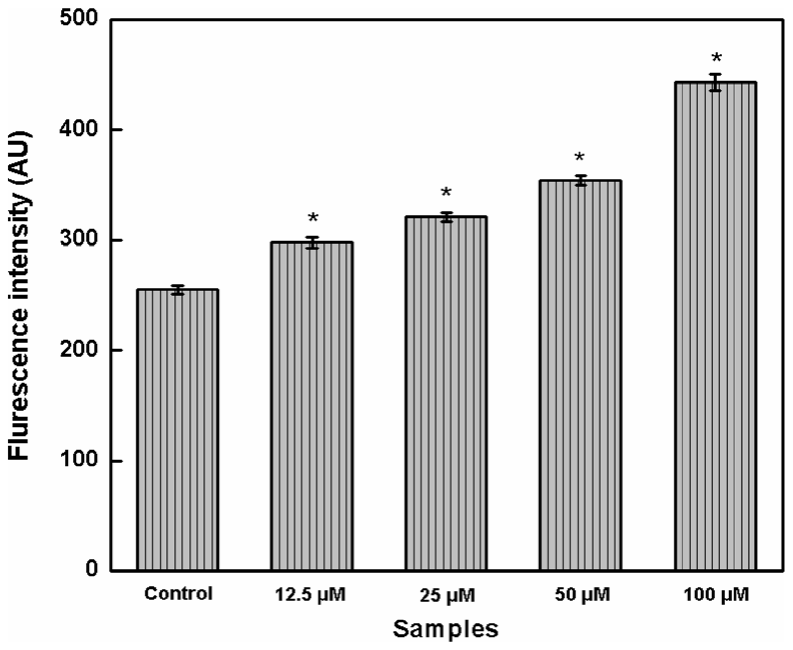
Histograms representing the intracellular ROS content in absence and presence of various concentrations (12.5–100 µM) of EGCG. (*p<0.05 as compared to control group).

The basal level of intracellular ROS in K562 cells (255.09±3.69 AU) is increased to 298.01±4.89, 320.83±4.03, 354.07±4.66, and 442.90±7.43 AU in presence of 12.5, 25, 50, and 100 µM concentration of EGCG (Figure 8).

## Discussion

Catechins are antioxidants ubiquitously found in green tea and among them EGCG shows highest antioxidant activity that may have therapeutic applications in cancer treatment. EGCG functions as a powerful antioxidant, preventing oxidative damage in healthy cells, but also as an anti-angiogenic and antitumor agent and as a modulator of tumor cell response to chemotherapy. However, neither pro-oxidant nor antioxidant activities have yet been clearly established to occur *in vivo* in humans [53]. Natural polyphenols are reported to be good inhibitors of human dihydrofolate reductase (DHFR) could explain the epidemiological data on their prophylactic effects for certain forms of cancer and open a possibility for the use of natural and synthetic polyphenols in cancer chemotherapy [54].

Fluorescence quenching experiments demonstrated that the ester bond containing tea polyphenols EGCG and ECG are effective inhibitors of DHFR with *K*_d_ of 0.9 and 1.8 µM, respectively, while polyphenols lacking the ester bond containing galloyl moiety (e.g., EGC and EC) did not bind to this enzyme [54]. The binding constant of EGCG to catalase was determined fluorimetrically to be 2.27×10^6^ M^−1^ (*K*_d_ of 0.44 µM). Other catechins were not as effective as EGCG establishing the influence of galloyl moiety for efficient binding. The binding efficiency is attributed to the eight phenolic groups of EGCG which serve as hydrogen bond donors. Involvement of positive charge on the carbon atom of the ester bond with the electronegative groups of amino acid residues like histidine also contributes towards efficient binding. Here it can be reasoned that at this concentration of EGCG it can also bind to other non-specific targets *in vivo* due to the presence of OH groups. To this end, it can be emphasized that binding to catalase inhibits its activity and increases the ROS population which eventually triggers apoptosis. It is known that 3-AT (an efficient inhibitor of catalase) binds with histidine residue (His75) near heme group of catalase forming a non-coplanar adduct (very close to Tyr358). Loss in fluorescence intensity of catalase suggests involvement of EGCG cation with histidine anion (p*K*a = 6.5) [55] near tyrosine residue (Tyr358) of active site [56]. This identification of the important structural features responsible for inhibition will provide valuable information for designing new inhibitors.

ITC provides valuable information on biomacromolecule-ligand interactions [57], [58]. This technique measures the heat released or absorbed (Δ*H*) when the macromolecule binds to ligands. This value of Δ*H* is used to determine other thermodynamic parameters. The thermodynamic parameters measured for the catalase-catechins interactions are summarized in Table 1. From the data, it is evident that the interactions of all four catechins with catalase are spontaneous and exothermic, which is confirmed by the negative values of Δ*G*. For all the catechins the favorable Δ*H* and unfavorable *T*Δ*S* values signify dominant forces of interaction to be hydrogen bonding with electrostatic contributions involving carbon cation of galloyl moiety. The catechins devoid of this group show lower affinity to catalase confirming the contribution of the cation towards electrostatic interactions with polar groups of the protein; which on binding are more exposed to interact with the galloyl moiety. The binding constant of EGCG with catalase determined from the thermodynamic parameters as 8.19×10^5^ M^−1^ using one-site binding model, is little lower than that obtained from fluorimetric measurements with one binding site. This is because the values observed in fluorescence spectroscopy are usually related to excited state complexes and ITC measures the ground state complexes. The binding constant for catalase-microcystin complex determined fluorimetrically is 6.12×10^4^ M^−1^ [40], which is lower than catalase-EGCG complex. Microcystin binding to catalase was reported to influence its physiological functions and conformation [40]. Binding is stronger with EGCG and more changes are observed in catalase-catechins complexes. Loss of α-helix is identified in the CD spectra by the decrease in negative peak at 222 nm and 208 nm (signature peaks of α-helix) on addition of EGCG as shown in Figure 5. Gain in β-sheet is observed from the increase in negative peak at 218 nm against 222 nm for α-helix. These conformational changes in catalase secondary structure on interaction with catechins influence its active site and physiological functions, rendering it inactive in scavenging ROS.

In the present study, the physiological changes are demonstrated by the inhibition of catalase activity by catechins. EGCG is observed to inhibit more efficiently than ECG and other non-gallated catechins. From the decay curves as shown in Figure 6, it is inferred that the rate of H_2_O_2_ degradation is reduced by catechins in cell free (Figure 6A) and cellular state (Figure 6B). From the Lineweaver-Burk plot (Figure 7), it is evident that the maximum inhibition of catalase is achieved by EGCG. The plot also demonstrates uncompetitive type of inhibition suggesting that EGCG binds to catalase at a site other than the active site. As a consequence, the uncompetitive inhibitor (here EGCG) lowers the measured maximum velocity (V_max_) and also decreases apparent K_m_ value [59]. This inhibition in catalase activity results in accumulation of ROS as illustrated in Figure 8. Higher levels of ROS create oxidative stress which is detrimental to cellular integrity.

The IC_50_ value for suppression of cell viability in K562 cells on EGCG treatment is determined to be 54.5 µM (or 27 µg/ml). Inhibition of human cancer cell growth by tea polyphenols has been observed in H1299, H661, HT-29, H441 and breast cancer cell lines at IC_50_ values between 20–75 µg/ml [39]. Higher EGCG concentration is required in the cell to elicit cellular response than its *K*_d_ value to catalase. Here it can be emphasized that suppression of cancer cell viability is not envisaged only by catalase inhibition but other pathways contribute to it. EGCG is capable of generating H_2_O_2_ in solution by auto-oxidation which contributes to oxidative stress. Superoxide is also reported to be generated in EGCG containing solutions [60]. In such conditions, inhibition of catalase by EGCG further increase the oxidative stress triggering apoptosis and eventually leading to cell death. These phenomena can be therapeutically used for cancer prevention. These revelations suggest that a balance between all the major activities of EGCG like antioxidants, pro-oxidants and enzyme inhibitors leads to cellular suppression, which is again cell specific. Inhibition of cellular enzymes by catechins has found major pharmacological applications as anticancer drugs [61]. Addition of methyl groups to ECG enhanced binding to DHFR and is a new approach to find novel inhibitors from natural resources [54]. A concentration of 27 µg/ml is high for EGCG to be bioavailable by tea consumption. Here EGCG is then treated as drug that can be delivered in various forms; one of which could be nano-formulations.

## Conclusion

The efficacy of EGCG to bind with catalase and render its activity in cancer cells definitely represents a fascinating tool in the field of oncology. The present work sheds light on the critical structural features of galloylated catechins and identifies probable mechanism by which they inhibit catalase. These findings provide guidance for designing efficient catalase inhibitors. However, a cancer cell death event is an endpoint of several pathways and further studies in this context are in progress to completely understand the role of catechins in anticancer therapy. This study also demonstrates that natural polyphenols could be used as ‘guide compounds’ for development of new anticancer drugs.

## References

[1] Trevisanato SI (2000) Tea and health. Nutr Rev 58: 1–10. 10697388 10.1111/j.1753-4887.2000.tb01818.x

[2] Crespy V, Williamson G (2004) A review of the health effects of green tea catechins in vivo animal models. J Nutr 134: 3431S–3440S. 15570050 10.1093/jn/134.12.3431S

[3] Siddiqui AI, Adhami VM, Saleem M, Mukhtar H (2006) Beneficial effects of tea and its polyphenols against prostate cancer. Mol Nutr Food Res 50: 130–143. 16425281 10.1002/mnfr.200500113

[4] Procházková D, Boušová I, Wilhelmová N (2011) Antioxidant and prooxidant properties of flavonoids. Fitoterapia 82: 513–523. 21277359 10.1016/j.fitote.2011.01.018

[5] Oikawa S, Furukawa A, Asada H, Hirakawa K, Kawanishi S (2003) Catechins induce oxidative damage to cellular and isolated DNA through the generation of reactive oxygen species. Free Radic Res 37: 881–890. 14567448 10.1080/1071576031000150751

[6] Halliwell B, Murcia A, Chirico S, Aruoma OI (1995) Free radicals and antioxidants in food and in vivo: what they do and how they work. Crit Rev Food Sci 35: 7–20. 10.1080/10408399509527682 7748482

[7] Yamashita N, Murata M, Inoue S, Burkitt MJ, Milne L, et al (1998) Alpha-tocopherol induces oxidative damage to DNA in the presence of copper (II) ions. Chem Res Toxicol 11: 855–862. 9705746 10.1021/tx970129v

[8] Yamashita N, Kawanishi S (2000) Distinct mechanisms of DNA damage in apoptosis induced by quercetin and luteolin. Free Radic Res 33: 623–633. 11200093 10.1080/10715760000301141

[9] Pal S, Saha C, Dey SK (2013) Studies on black tea (*Camellia sinensis*) extract as a potential antioxidant and a probable radioprotector. Radiat Environ Biophys 52: 269–278. 23519756 10.1007/s00411-013-0463-z

[10] Ghosh D, Pal S, Saha C, Chakrabarti AK, Datta SC, et al (2012) Black tea extract: a supplementary antioxidant in radiation induced damage to DNA and normal lymphocytes. J Environ Pathol Toxicol Oncol 31: 1–12. 23216640 10.1615/jenvironpatholtoxicoloncol.v31.i2.70

[11] Higdon JV, Frei B (2003) Tea catechins and polyphenols: health effects, metabolism, and antioxidant functions. Crit Rev Food Sci 43: 89–143. 10.1080/10408690390826464 12587987

[12] Richi B, Kale RK, Tiku AB (2012) Radio-modulatory effects of green tea catechin EGCG on pBR322 plasmid DNA and murine splenocytes against gamma-radiation induced damage. Mutat Res 747: 62–70. 22521723 10.1016/j.mrgentox.2012.04.002

[13] Nair CKK, Salvi VP (2008) Protection of DNA from gamma-radiation induced strand breaks by epicatechin. Mutat Res 650: 48–54. 18006366 10.1016/j.mrgentox.2007.10.001

[14] Yang GY, Liao J, Kim K, Yurkow EJ, Yang CS (1998) Inhibition of growth and induction of apoptosis in human cancer cell lines by tea polyphenols. Cacinogenesis 19: 611–616. 10.1093/carcin/19.4.611 9600345

[15] Cao YH, Cao RH (1999) Angiogenesis inhibited by drinking tea. Nature 398: 381. 10201368 10.1038/18793

[16] Ahmad N, Feyes DK, Nieminen AL, Agarwal R, Mukhtar H (1997) Green tea constituent epigallocatechin-3-gallate and induction of apoptosis and cell cycle arrest in human carcinoma cells. J Natl Cancer Inst 89: 1881–1886. 9414176 10.1093/jnci/89.24.1881

[17] Lyn-Cook BD, Rogers T, Yan Y, Blann EB, Kadlubar FF, et al (1990) Chemopreventive effects of tea extracts and various components on human pancreatic and prostate tumor cells *in vitro* . Nutr Cancer 35: 80–86. 10.1207/S1532791480-86 10624710

[18] Yang CS, Wang X, Lu G, Picinich SC (2009) Cancer prevention by tea: animal studies, molecular mechanisms and human relevance. Nat Rev Cancer 9: 429–439. 19472429 10.1038/nrc2641 PMC2829848

[19] Ishii T, Ishikawa M, Miyoshi N, Yasunaga M, Akagawa M, et al (2009) Catechol type polyphenol is a potential modifier of protein sulfhydryls: development and application of a new probe for understanding the dietary polyphenol actions. Chem Res Toxicol 22: 1689–1698. 19743802 10.1021/tx900148k

[20] Ishii T, Mori T, Tanaka T, Mizuno D, Yamaji R, et al (2008) Covalent modification of proteins by green tea polyphenol (−)-epigallocatechin-3-gallate through autoxidation. Free Radic Biol Med 45: 1384–1394. 18771724 10.1016/j.freeradbiomed.2008.07.023

[21] Wang X, Song KS, Guo QX, Tian WX (2003) The galloyl moiety of green tea catechins is the critical structural feature to inhibit fatty-acid synthase. Biochem Pharmacol 66: 2039–2047. 14599562 10.1016/s0006-2952(03)00585-9

[22] Sadik CD, Sies H, Schewe T (2003) Inhibition of 15-lipoxygenases by flavonoids: structure-affinity relations and mode of action. Biochem Pharmacol 65: 773–781. 12628491 10.1016/s0006-2952(02)01621-0

[23] Sazuka M, Itoi T, Suzuki Y, Odani S, Koide T, et al (1996) Evidence for the interaction between (−)-epigallocatechin gallate and human plasma proteins fibronectin, fibrinogen, and histidine-rich glycoprotein. Biosci Biotechnol Biochem 60(8): 1317–1319. 8987550 10.1271/bbb.60.1317

[24] Dubeau S, Samson G, Tajmir-Riahi HA (2010) Dual effect of milk on the antioxidant capacity of green, Darjeeling, and English breakfast teas. Food Chem 122: 539–545.

[25] Hasni I, Bourassa P, Hamdani S, Samson G, Carpentier R, et al (2011) Interaction of milk α- and β-caseins with tea polyphenols. Food Chem 126: 630–639.

[26] Kanakis CD, Hasni I, Bourassa P, Tarantilis PA, Polissiou MG (2011) Milk β-lactoglobulin complexes with tea polyphenols. Food Chem 127: 1046–1055. 25214095 10.1016/j.foodchem.2011.01.079

[27] Bourassa P, Cote R, Hutchandani S, Samson G, Tajmir-Riahi HA (2013) The effect of milk alpha-casein on the antioxidant activity of tea polyphenols. J Photochem Photobiol B 128: 43–49. 24001682 10.1016/j.jphotobiol.2013.07.021

[28] Leone M, Zhai D, Sareth S, Kitada S, Reed JC, et al (2003) Cancer prevention by tea polyphenols is linked to their direct inhibition of antiapoptotic bcl-2-family proteins. Cancer Res 63: 8118–8121. 14678963

[29] Ermakova S, Choi BY, Choi HS, Kang BS, Bode AM, et al (2005) The intermediate filament protein vimentin is a new target for epigallocatechin gallate. J Biol Chem 280: 16882–16890. 15713670 10.1074/jbc.M414185200

[30] Shim JH, Su ZY, Chae JI, Kim DJ, Zhu F, et al (2010) Epigallocatechin gallate suppresses lung cancer cell growth through Ras-GTPase-activating protein SH3 domain-binding protein 1. Cancer Prev Res 3: 670–679. 10.1158/1940-6207.CAPR-09-0185 20424128

[31] Deisseroth A, Dounce AL (1970) Catalase: Physical and chemical properties, mechanism of catalysis, and physiological role. Physiol Rev 50: 319–375. 4912904 10.1152/physrev.1970.50.3.319

[32] Percy ME (1984) Catalase: an old enzyme with a new role? Can J Biochem Cell Biol 62: 1006–1014. 6095974 10.1139/o84-129

[33] Sund H, Weber K, Molbert E (1967) Dissociation of beef liver catalase in its subunits, Eur J Biochem. 1: 400–410. 10.1111/j.1432-1033.1967.tb00088.x 6061959

[34] Stern KG (1936) The constitution of the prosthetic group of catalase. J Biol Chem 112: 661–669.

[35] Yang L, Zheng XL, Sun H, Zhong YZ, Wang Q, et al (2011) Catalase suppression-mediated H_2_O_2_ accumulation in cancer cells by wogonin effectively blocks tumor necrosis factor-induced NF-κB activation and sensitizes apoptosis. Cancer Sci 102: 870–876. 21244577 10.1111/j.1349-7006.2011.01874.x

[36] Dong Z, Ma W, Huang C, Yang CS (1997) Inhibition of tumor promoter-induced activator protein 1 activation and cell transformation by tea polyphenols, (−)-epigallocatechin gallate, and theaflavins. Cancer Res 57: 4414–4419. 9331105

[37] Liang YC, Lin-Shiau SY, Chen CF, Lin JK (1999) Inhibition of cyclin-dependent kinases 2 and 4 activities as well as induction of Cdk inhibitors p21 and p27 during growth arrest of human breast carcinoma cells by (-)-epigallocatechin-3-gallate. J Cell Biochem 75: 1–12. 10462699

[38] Fang MZ, Wang Y, Ai N, Hou Z, Sun Y, et al (2003) Tea polyphenol (-)-epigallocatechin-3-gallate inhibits DNA methyltransferase and reactivates methylation-silenced genes in cancer cell lines. Cancer Res 63: 7563–7570. 14633667

[39] Yang G, Liao J, Kim K, Yurkow EJ, Yang CS (1998) Inhibition of growth and induction of apoptosis in human cancer cell lines by tea polyphenols. Carcinogenesis 19: 611–616. 9600345 10.1093/carcin/19.4.611

[40] Hu Y, Da L (2014) Insights into the selective binding and toxic mechanism of microcystin to catalase. Spectrochim Acta A Mol Biomol Spectrosc 121: 230–237. 24247095 10.1016/j.saa.2013.09.078

[41] Lenehan PF, Gutiérrez PL, Wagner JL, Milak N, Fisher GR, et al (1995) Resistance to oxidants associated with elevated catalase activity in HL-60 leukemia cells that overexpress multidrug-resistance protein does not contribute to daunorubicin manifested by these cells. Cancer Chemother Pharmacol 35: 377–386. 7850918 10.1007/s002800050250

[42] Bechtel W, Bauer G (2009) Catalase protects tumor cells from apoptosis induction by intracellular ROS signaling. Anticancer Res 29: 4541–4557. 20032403

[43] Liu WB, Zhou J, Qu Y, Li X, Lu CT, et al (2010) Neuroprotective effect of osthole on MPP^+^-induced cytotoxicity in PC12 cells via inhibition of mitochondrial dysfunction and ROS production. Neurochem Int 57: 206–215. 20510317 10.1016/j.neuint.2010.05.011

[44] Attar F, Khavari-Nejad S, Keyhani J, Keyhani E (2009) Structural and functional alterations of catalase induced by acriflavine, a compound causing apoptosis and necrosis. Ann New York Acad Sci 1171: 292–299. 19723068 10.1111/j.1749-6632.2009.04683.x

[45] Jelesarov I, Bosshard HR (1999) Isothermal titration calorimetry and differential scanning calorimetry as complementary tools to investigate the energetics of biomolecular recognition. J Mol Recognit 12: 3–18. 10398392 10.1002/(SICI)1099-1352(199901/02)12:1<3::AID-JMR441>3.0.CO;2-6

[46] Wiseman T, Williston S, Brands JF, Lin LN (1989) Rapid measurements of binding constants and heats of binding using titration calorimeter. Anal Biochem 179: 131–137. 2757186 10.1016/0003-2697(89)90213-3

[47] Pal S, Saha C, Hossain M, Dey SK, Kumar GS (2012) Influence of galloyl moiety in interaction of epicatechin with bovine serum albumin: a spectroscopic and thermodynamic characterization. Plos One 7(8): e43321 doi:10.1371/journal.pone.0043321 22916242 10.1371/journal.pone.0043321 PMC3423357

[48] Jha NS, Kishore N (2009) Binding of streptomycin with bovine serum albumin: energetics and conformational aspects. Thermochim Acta 482: 21–29.

[49] Hossain M, Khan AY, Kumar GS (2011) Interaction of the anticancer plant alkaloid sanguinarine with bovine serum albumin. Plos One 6(4): e18333 doi:10.1371/journal.pone.0018333 21494677 10.1371/journal.pone.0018333 PMC3071820

[50] Khan MA, Muzammil S, Musarrat J (2002) Differential binding of tetracyclines with serum albumin and induced structural alterations in drug-bound protein. Int J Biol Macromol 30: 243–249. 12297231 10.1016/s0141-8130(02)00038-7

[51] Liu JQ, Tian JN, He WY, Xie JP, Hu ZD, et al (2004) Spectrofluorimetric study of the binding of daphnetin to bovine serum albumin. J Pharm Biomed Anal 35: 671–677. 15137995 10.1016/j.jpba.2004.02.010

[52] Ashoka S, Seetharamappa J, Kandagal PB, Shaikh SMT (2006) Investigation of the interaction between trazodone hydrochloride and bovine serum albumin. J Lumin 121: 179–186. 10.1016/j.ijbiomac.2006.03.027 16678251

[53] Halliwell B (2008) Are polyphenols antioxidants or pro-oxidants? What do we learn from cell culture and in vivo studies? Arch Biochem Biophys 476: 107–112. 18284912 10.1016/j.abb.2008.01.028

[54] Sánchez-del-Campo L, Sáez-Ayala M, Chazarra S, Cabezas-Herrera J, Rodríguez-López JN (2009) Binding of natural and synthetic polyphenols to human dihydrofolate reductase. Int J Mol Sci 10: 5398–5410. 20054477 10.3390/ijms10125398 PMC2802001

[55] Al-Shakhshir R, Regnier F, White JL, Hem SL (1994) Effect of protein adsorption on the surface charge characteristics of aluminium-containing adjuvants. Vaccine 5: 472–474. 10.1016/0264-410x(94)90127-9 8023556

[56] Putnam CD, Arvai AS, Bourne Y, Tainer JA (2000) Active and inhibited human catalase structures: ligand and NADPH binding and catalytic mechanism. J Mol Biol 296: 295–309. 10656833 10.1006/jmbi.1999.3458

[57] Pierce MM, Raman CS, Nall BT (1999) Isothermal titration calorimetry of protein-protein interactions. Methods 19: 213–221. 10527727 10.1006/meth.1999.0852

[58] Gohlke H, Klebe G (2002) Approaches to the description and prediction of the binding affinity of small-molecule ligands to macromolecular receptors. Anqew Chem Int Ed Enql 41: 2644–2676. 10.1002/1521-3773(20020802)41:15<2644::AID-ANIE2644>3.0.CO;2-O 12203463

[59] Nelson DL, Cox MM (2004) Lehninger Principles of Biochemistry. Enzymes, Freeman. 190–211.

[60] Elbling L, Weiss RM, Teufelhofer O, Uhl M, Knasmueller S, et al (2005) Green tea extract and (−)-epigallocatechin-3-gallate, the major tea catechin, exert oxidant but lack antioxidant activities. FASEB J 19: 807–809. 15738004 10.1096/fj.04-2915fje

[61] Trachootham D, Alexandre J, Huang P (2009) Targeting cancer cells by ROS mediated mechanisms: a radical therapeutic approach? Nat Rev Drug Discov 8: 579–591. 19478820 10.1038/nrd2803

